# Supplementary material for: Spatially resolved gene expression analysis illuminates location-specific functions in the reef-building coral Pocillopora acuta
Source: PLoS One. 2026 Sep 16;21(9):e0358454. doi: 10.1371/journal.pone.0358454 (PMC13581030; doi:10.1371/journal.pone.0358454)
Supplement: Supplementary file 1 [file pone.0358454.s014.docx]

*PLOS One*	August 28, 2026

Dear Editor and Reviewers,

Thank you for taking the time to review our manuscript entitled “Spatially resolved gene expression analysis illuminates location-specific functions in the reef-building coral *Pocillopora acuta*”. Included with this letter you will find a point-by-point response to the reviewer’s comments.

We appreciate the comments and have included revisions and responses to each comment and suggestion.

Our updated funding statement is: “The LCM instrumentation for this work was provided by a Champlin Foundation grant to the URI College of Pharmacy and College of the Environment and Life Sciences. The cryostat used in this work was provided by funding from an NIH Instrumentation award 1S10OD032209-01. This work was also supported by an internal URI College of the Environment and Life Sciences grant and an external NSF Division of Biological Infrastructure grant 2316390 to HMP. This material is based upon work supported by the National Science Foundation Graduate Research Fellowship Program under Grant No 2146759 to ZD. Any opinions, findings, and conclusions or recommendations expressed in this material are those of the author(s) and do not necessarily reflect the views of the National Science Foundation. *There was no additional external funding received for this study.”*

Our updated Role of Funder statement is: “*The funders had no role in study design, data collection and analysis, decision to publish, or preparation of the manuscript.*”

Please contact us should you require any further information.

Sincerely,

Zoe Dellaert and Hollie Putnam

Reviewers' comments (Our responses in blue text):

**Reviewer #1:** The manuscript by Dellaert and Putnam reports a transcriptomic comparison between oral and aboral territories in apolyps of a reef-building coral species, Pocillopora acuta. This bulk transcriptomic approach complements recent efforts to characterize the cellular repertoire in cnidarians, a group of major interest to understand the origins of bilaterians.

The results from the transcriptomic analysis are robust, well described, well illustrated by quality figures. They are adequately analyzed in terms of functional annotation, with a focus on processes of interest: metabolism, development, response to biotic or abiotic stimuli. As such, they provide a reference for further studies.

As a non-specialist of the field, I have only minor suggestions for improvement:

Experimental aspects:

The work would be valued by direct ISH or HCR-ISH validations of the transcriptomic data, focussing on processes of interest.

We agree that the targeted quantification of expression is the a powerful approach, but given the biological validation we have presented in our work and the point of our paper providing a whole transcriptome view of targeted tissues, the use of direct ISH or HCR-ISH validations of the transcriptomic data would represent a substantial transition of focus and investment of time and money that is beyond the scope of what is available to this work To acknowledge the value and power of direct ISH or HCR-ISH data we have highlighted where our study has the support of multiple studies for biological validation (Lines 661, 698, 711, 723, 729) and we have also added a statement regarding this to the discussion as a recommendation for future work: “Future studies validating these findings with *in situ hybridization* approaches (e.g., HCR-ISH), particularly for the non-canonical expression of developmental signaling pathways and genes with unexpected tissue-level expression patterns, will greatly advance our understanding of tissue-specific function in corals.” (Line 745)

Minor text modifications:

1. A phylogenetic tree showing the relationships between cnidarians, with the relative position of P. acuta and other species submitted to scRNA-seq analysis or other transcriptomic approach would help the non-specialist reader to have a broader view of data available in this taxon

If the reviewer deems this suitable, we have added the following phylogenetic tree to the supplement (S1 Fig). We do note that the scRNA marker genes referred to in our main text are all from Levy et al. 2021, which is in a closely related species of the same Pocilloporidae family: “, we examined the expression of scRNA-seq marker genes from another Pocilloporid species: *S. pistillata* [31].” (Line 539).

2. Throughout the manuscript, the authors tend to oversell the spatial resolution brought by their analysis: these data provide a comparison between two spatially distinct tissues. But spatially resolved transcriptomics rely on completely different approaches and provide much more detailed information.

We agree that there are now novel high resolution approaches available at the whole transcriptome level (e.g., spatial transcriptomics and work at the single cell and single nuclei). This is stated in the abstract (Line 47) and we have now added text to reinforce our recommendations for future work in the conclusion: “Sequencing-based methods with higher spatial resolution than the tissue-level work in this paper (e.g., spatial transcriptomics) will be crucial to fully resolve the tissue complexity of these organisms.” (Line 749).

While the novel spatial transcriptomics methods can provide higher resolution and cell-specific information than the approach in our manuscript, our application of Laser Capture Microdissection offers a more affordable alternative for studying the tissues of these organisms, enabling higher replication and broader accessibility to research groups.

The introduction has been revised to not oversell the resolution of LCM (Line 140).

3. line 49 "Corals are complex symbiotic meta-organisms with functionally partitioned tissue architecture,thus integrating tissue-specific processes is essential for improving predictions of responses to climate change stressors and identifying mechanisms underlying resilience": truncated sentence?

We have revised it to read: “Corals are complex symbiotic meta-organisms with functionally partitioned tissue architecture. Thus, integrating tissue-specific processes is essential for improving predictions of responses to climate change stressors and identifying mechanisms underlying their resilience.” (Line 66)

4. line 477 "Both oral and aboral tissues respond to biotic and abiotic stimulus": this is not demonstrated, even if the data support the hypothesis, and it should be reformulated.

We have revised this heading to be more specific: “Both oral and aboral tissues express biotic and abiotic stimulus-response genes” (Line 519)

**Reviewer #2:** In this manuscript, Dellaert and Putnam investigate genes that are differentially expressed between the oral and aboral sides of the reef-building coral Pocillopora acuta. To do so, they performed RNA sequencing on samples obtained from polyp cryosections using laser capture microdissection. This method is novel in corals, and the resulting data appear to be of high quality, providing a unique dataset for analyzing differential gene expression between the oral and aboral sides of coral polyps. Based on these analyses, the authors draw several interesting conclusions regarding the localization of previously identified cell types and the possible role of Wnt signaling in skeleton formation on the aboral side. I have only a few minor comments that I would like the authors to address:

When analyzing genes that are differentially expressed between the oral and aboral sides of the polyp, the authors rely heavily on Gene Ontology (GO) terms. While this leads to some interesting conclusions, it also results in the discussion of GO terms that seem irrelevant for cnidarians, such as "skin morphogenesis" (L465) and "oviduct epithelium development" (L466), as well as others whose relevance is far from straightforward in cnidarians, including "stimulus involved in sensory perception," "detection of abiotic stimulus," "response to norepinephrine," "cellular response to thyroid hormone stimulus," and "complement activation, classical pathway" (L478–488). I would suggest restricting the discussion to GO terms that are likely, or at least plausibly, applicable to cnidarians.

Because GO terms are primarily derived from model organisms such as humans and *C. elegans*, some terms can appear inapplicable to cnidarians at face value. However, the underlying genes assigned to these terms often represent deeply conserved evolutionary pathways that are highly relevant to cnidarian biology. For example, cnidarians have sensory cells that respond to touch, light, and chemical cues, and cnidarian innate immunity is crucial to active regulation of endosymbiont populations via digestion and apoptosis induction. These terms mentioned by the reviewer, therefore despite the arguable relevance of their term name, capture biologically meaningful signals in our dataset, even if the human-derived term labels are not literal descriptions of cnidarian processes. Therefore we have maintained all of the significantly enriched GO terms as they identify groups of genes under a common process that are enriched and should be examined for their cnidarian specific biology, not thrown out.

To make this framework explicit, we have added the statement following to the Methods: “We acknowledge that many GO terms are labelled based on mammalian- and human-specific processes, but the specific genes underlying the enrichment of certain terms represent shared evolutionary pathways that are relevant to cnidarian biology. Throughout the text, we use GO terms as tools to identify broad patterns of genes, but all biological interpretation is based on the genes themselves, not the GO term labels.” (Line 344).

In response to the reviewer’s suggestion, we have also edited Figure 3 to replace the term “camera-type eye development” with its parent term “sensory system development,” which more accurately reflects the context of our study. We have indicated this by an * after the term and stated this in the figure caption. Where other term labels appear model-system-specific, we have provided both the label and the parent term label to indicate the broader processes involved (Lines 504, 506).

L473–474: "The top five upregulated genes contributing to these enrichments." It is unclear what "these" refers to. Please clarify.

Thank you for pointing this out! We have revised the sentence to: “The top five upregulated genes contributing to the enrichments of GO terms in of Oral Clusters 1-3 were: *Transcription factor Sox2, Peroxidasin homolog pxn-2, Wnt-2b, Double-stranded RNA-specific editase 1,* and *Dynein axonemal heavy chain 5*.” (Line 514)

L537–542: The authors report stronger GO term enrichment on the aboral side but a greater number of differentially expressed genes on the oral side. They conclude that this suggests "transcriptional diversity rather than coordination" on the oral side, whereas the aboral side exhibits "a functionally coordinated expression profile." I do not fully understand what the authors mean by this interpretation. I suggest rewriting this section to make the rationale clearer.

This has been revised to read “While oral tissues showed substantially more upregulated genes than aboral tissue, GO enrichment revealed relatively few oral-enriched functional categories, suggesting that many diverse genes with different functions were upregulated. In contrast, aboral tissues showed fewer upregulated genes, but a larger number of significantly enriched GO terms, indicating that the upregulated genes shared similar functions.” (Line 587)

L471–472: Among the genes enriched on the aboral side of the polyp, the authors identify several genes known to be expressed on the aboral side of cnidarian planulae, including Frizzled5, SFRP1/5, and SFRP3/4, as reported in Ref. 42. Are other conserved aboral genes identified in Ref. 42, such as Six3/6, Hox9–14, and DKK, also present in this dataset? This is an interesting observation that could be further discussed in the Discussion.

We have added a reference to the Wnt signalling expression patterns documented in larvae by Ref. 42 in the Discussion (Line 698). We note that in the supplementary materials of Ref. 42 we are not able to find sufficient information to unambiguously identify gene IDs to search for orthologs based on the shortened gene names used in the text of Ref. 42. We therefore examined the expression of a series of *Nematostella* Wnt and Hox genes described in Ref. 172 (DuBuc et al., 2018) and found that these overlapped with the genes already discussed in our Results and Discussion; no additional text was added to the Discussion regarding these genes.

Of the additional specific genes from Ref. 42 mentioned by the reviewer:

- *Six3/6* was upregulated in aboral tissue but did not meet the cutoff of |LFC| > 1 (log2FoldChange = -0.90, padj = 0.026).
- *Dkk1/2/4* was not expressed in our study.
- *Hox9-14* does not appear to have a clearly annotated *Pocillopora* ortholog in Ref. 42, and we were unable to identify a Hox9-14 ortholog in our expressed genes.
