# Supplementary material for: Spatially resolved gene expression analysis illuminates location-specific functions in the reef-building coral Pocillopora acuta
Source: PLoS One. 2026 Sep 16;21(9):e0358454. doi: 10.1371/journal.pone.0358454 (PMC13581030; doi:10.1371/journal.pone.0358454)
Supplement: Peer review history

# Peer review: Decision Letter 0

**DOI:** 10.1371/journal.pone.0358454.r001

**Authors:** Hector Escriva

20 Jul 2026

*Pocillopora acuta*

Dear Dr. Dellaert,

Thank you for submitting your manuscript to PLOS ONE. After careful consideration, we feel that it has merit but does not fully meet PLOS ONE’s publication criteria as it currently stands. Therefore, we invite you to submit a revised version of the manuscript that addresses the points raised during the review process.

Both reviewers found the work interesting and recognized its potential contribution. However, they also identified a number of mistakes and points requiring clarification or correction. We therefore invite you to revise the manuscript by carefully addressing all of the reviewers' comments and incorporating the necessary changes before resubmitting your revised version for further consideration.

Please submit your revised manuscript by Sep 03 2026 11:59PM. If you will need more time than this to complete your revisions, please reply to this message or contact the journal office at plosone@plos.org. When you're ready to submit your revision, log on to https://www.editorialmanager.com/pone/ and select the 'Submissions Needing Revision' folder to locate your manuscript file.

- A letter that responds to each point raised by the academic editor and reviewer(s). You should upload this letter as a separate file labeled 'Response to Reviewers'.
- A marked-up copy of your manuscript that highlights changes made to the original version. You should upload this as a separate file labeled 'Revised Manuscript with Track Changes'.
- An unmarked version of your revised paper without tracked changes. You should upload this as a separate file labeled 'Manuscript'.

If you would like to make changes to your financial disclosure, please include your updated statement in your cover letter. Guidelines for resubmitting your figure files are available below the reviewer comments at the end of this letter.

If applicable, we recommend that you deposit your laboratory protocols in protocols.io to enhance the reproducibility of your results. Protocols.io assigns your protocol its own identifier (DOI) so that it can be cited independently in the future. For instructions see: https://journals.plos.org/plosone/s/submission-guidelines#loc-laboratory-protocols. Additionally, PLOS ONE offers an option for publishing peer-reviewed Lab Protocol articles, which describe protocols hosted on protocols.io. Read more information on sharing protocols at https://plos.org/protocols?utm_medium=editorial-email&utm_source=authorletters&utm_campaign=protocols.

As the corresponding author, your ORCID iD is verified in the submission system and will appear in the published article. PLOS supports the use of ORCID, and we encourage all coauthors to register for an ORCID iD and use it as well. Please encourage your coauthors to verify their ORCID iD within the submission system before final acceptance, as unverified ORCID iDs will not appear in the published article. *Only*  the individual author can complete the verification step; PLOS staff *cannot*  verify ORCID iDs on behalf of authors.

We look forward to receiving your revised manuscript.

Kind regards,

Hector Escriva, PhD

Academic Editor

PLOS One

Journal Requirements:

When submitting your revision, we need you to address these additional requirements.

1. Please ensure that your manuscript meets PLOS ONE's style requirements, including those for file naming. The PLOS ONE style templates can be found at

https://journals.plos.org/plosone/s/file?id=wjVg/PLOSOne_formatting_sample_main_body.pdf and

https://journals.plos.org/plosone/s/file?id=ba62/PLOSOne_formatting_sample_title_authors_affiliations.pdf.

2. Thank you for stating in your Funding Statement:

“This work was also supported by funding from an NIH Instrumentation award 1S10OD032209-01, URI College of the Environment and Life Sciences Internal grant, and NSF Division of Biological Infrastructure grant 2316390 to HMP. This material is based upon work supported by the National Science Foundation Graduate Research Fellowship Program under Grant No 2146759 to ZD. “

Please provide an amended statement that declares *all* the funding or sources of support (whether external or internal to your organization) received during this study, as detailed online in our guide for authors at http://journals.plos.org/plosone/s/submit-now.  Please also include the statement “There was no additional external funding received for this study.” in your updated Funding Statement.

Please include your amended Funding Statement within your cover letter. We will change the online submission form on your behalf.

3. Thank you for stating the following financial disclosure:

“This work was also supported by funding from an NIH Instrumentation award 1S10OD032209-01, URI College of the Environment and Life Sciences Internal grant, and NSF Division of Biological Infrastructure grant 2316390 to HMP. This material is based upon work supported by the National Science Foundation Graduate Research Fellowship Program under Grant No 2146759 to ZD. “

Please state what role the funders took in the study.  If the funders had no role, please state: "The funders had no role in study design, data collection and analysis, decision to publish, or preparation of the manuscript."

If this statement is not correct you must amend it as needed.

Please include this amended Role of Funder statement in your cover letter; we will change the online submission form on your behalf.

4.Thank you for stating the following in the Acknowledgments Section of your manuscript:

“This work was also supported by funding from an NIH Instrumentation award 1S10OD032209-01, URI College of the Environment and Life Sciences Internal grant, and NSF Division of Biological Infrastructure grant 2316390 to HMP. This material is based upon work supported by the National Science Foundation Graduate Research Fellowship Program under Grant No 2146759”

We note that you have provided additional information within the Acknowledgements Section that is not currently declared in your Funding Statement. Please note that funding information should not appear in the Acknowledgments section or other areas of your manuscript. We will only publish funding information present in the Funding Statement section of the online submission form.

Please remove any funding-related text from the manuscript and let us know how you would like to update your Funding Statement. Currently, your Funding Statement reads as follows:

“This work was also supported by funding from an NIH Instrumentation award 1S10OD032209-01, URI College of the Environment and Life Sciences Internal grant, and NSF Division of Biological Infrastructure grant 2316390 to HMP. This material is based upon work supported by the National Science Foundation Graduate Research Fellowship Program under Grant No 2146759 to ZD. “

Please include your amended statements within your cover letter; we will change the online submission form on your behalf.

5. If the reviewer comments include a recommendation to cite specific previously published works, please review and evaluate these publications to determine whether they are relevant and should be cited. There is no requirement to cite these works unless the editor has indicated otherwise.

6. Please review your reference list to ensure that it is complete and correct. If you have cited papers that have been retracted, please include the rationale for doing so in the manuscript text, or remove these references and replace them with relevant current references. Any changes to the reference list should be mentioned in the rebuttal letter that accompanies your revised manuscript. If you need to cite a retracted article, indicate the article’s retracted status in the References list and also include a citation and full reference for the retraction notice.

[Note: HTML markup is below. Please do not edit.]

Reviewers' comments:

Reviewer's Responses to Questions

**Comments to the Author**

1. Is the manuscript technically sound, and do the data support the conclusions?

Reviewer #1: Yes

Reviewer #2: Yes

**********

2. Has the statistical analysis been performed appropriately and rigorously?

Reviewer #1: Yes

Reviewer #2: Yes

**********

3. Have the authors made all data underlying the findings in their manuscript fully available?

The PLOS Data policy

Reviewer #1: Yes

Reviewer #2: Yes

**********

4. Is the manuscript presented in an intelligible fashion and written in standard English?

Reviewer #1: Yes

Reviewer #2: Yes

**********

Reviewer #1: The manuscript by Dellaert and Putnam reports a transcriptomic comparison between oral and aboral territories in apolyps of a reef-building coral species, Pocillopora acuta. This bulk transcriptomic approach complements recent efforts to characterize the cellular repertoire in cnidarians, a group of major interest to understand the origins of bilaterians.

The results from the transcriptomic analysis are robust, well described, well illustrated by quality figures. They are adequately analyzed in terms of functional annotation, with a focus on processes of interest: metabolism, development, response to biotic or abiotic stimuli. As such, they provide a reference for further studies.

As a non-specialist of the field, I have only minor suggestions for improvement:

Experimental aspects:

The work would be valued by direct ISH or HCR-ISH validations of the transcriptomic data, focussing on processes of interest.

Minor text modifications:

1. A phylogenetic tree showing the relationships between cnidarians, with the relative position of P. acuta and other species submitted to scRNA-seq analysis or other transcriptomic approach would help the non-specialist reader to have a broader view of data available in this taxon

2. Throughout the manuscript, the authors tend to oversell the spatial resolution brought by their analysis: these data provide a comparison between two spatially distinct tissues. But spatially resolved transcriptomics rely on completely different approaches and provide much more detailed information.

3. line 49 "Corals are complex symbiotic meta-organisms with functionally partitioned tissue architecture,thus integrating tissue-specific processes is essential for improving predictions of responses to climate change stressors and identifying mechanisms underlying resilience": truncated sentence?

4. line 477 "Both oral and aboral tissues respond to biotic and abiotic stimulus": this is not demonstrated, even if the data support the hypothesis, and it should be reformulated.

Reviewer #2: In this manuscript, Dellaert and Putnam investigate genes that are differentially expressed between the oral and aboral sides of the reef-building coral Pocillopora acuta. To do so, they performed RNA sequencing on samples obtained from polyp cryosections using laser capture microdissection. This method is novel in corals, and the resulting data appear to be of high quality, providing a unique dataset for analyzing differential gene expression between the oral and aboral sides of coral polyps. Based on these analyses, the authors draw several interesting conclusions regarding the localization of previously identified cell types and the possible role of Wnt signaling in skeleton formation on the aboral side. I have only a few minor comments that I would like the authors to address:

When analyzing genes that are differentially expressed between the oral and aboral sides of the polyp, the authors rely heavily on Gene Ontology (GO) terms. While this leads to some interesting conclusions, it also results in the discussion of GO terms that seem irrelevant for cnidarians, such as "skin morphogenesis" (L465) and "oviduct epithelium development" (L466), as well as others whose relevance is far from straightforward in cnidarians, including "stimulus involved in sensory perception," "detection of abiotic stimulus," "response to norepinephrine," "cellular response to thyroid hormone stimulus," and "complement activation, classical pathway" (L478–488). I would suggest restricting the discussion to GO terms that are likely, or at least plausibly, applicable to cnidarians.

L473–474: "The top five upregulated genes contributing to these enrichments." It is unclear what "these" refers to. Please clarify.

L537–542: The authors report stronger GO term enrichment on the aboral side but a greater number of differentially expressed genes on the oral side. They conclude that this suggests "transcriptional diversity rather than coordination" on the oral side, whereas the aboral side exhibits "a functionally coordinated expression profile." I do not fully understand what the authors mean by this interpretation. I suggest rewriting this section to make the rationale clearer.

L471–472: Among the genes enriched on the aboral side of the polyp, the authors identify several genes known to be expressed on the aboral side of cnidarian planulae, including Frizzled5, SFRP1/5, and SFRP3/4, as reported in Ref. 42. Are other conserved aboral genes identified in Ref. 42, such as Six3/6, Hox9–14, and DKK, also present in this dataset? This is an interesting observation that could be further discussed in the Discussion.

**********

what does this mean?). If published, this will include your full peer review and any attached files.

If you choose “no”, your identity will remain anonymous but your review may still be made public.

**Do you want your identity to be public for this peer review?**  For information about this choice, including consent withdrawal, please see our Privacy Policy

Reviewer #1: No

Reviewer #2: No

**********

[NOTE: If reviewer comments were submitted as an attachment file, they will be attached to this email and accessible via the submission site. Please log into your account, locate the manuscript record, and check for the action link "View Attachments". If this link does not appear, there are no attachment files.]

To ensure your figures meet our technical requirements, please review our figure guidelines: https://journals.plos.org/plosone/s/figures

You may also use PLOS’s free figure tool, NAAS, to help you prepare publication quality figures: https://journals.plos.org/plosone/s/figures#loc-tools-for-figure-preparation.

NAAS will assess whether your figures meet our technical requirements by comparing each figure against our figure specifications.

# Author comment: Author response to Decision Letter 1

**DOI:** 10.1371/journal.pone.0358454.r002

28 Aug 2026

PLOS One August 28, 2026

Dear Editor and Reviewers,

Thank you for taking the time to review our manuscript entitled “Spatially resolved gene expression analysis illuminates location-specific functions in the reef-building coral Pocillopora acuta”. Included with this letter you will find a point-by-point response to the reviewer’s comments.

We appreciate the comments and have included revisions and responses to each comment and suggestion.

Our updated funding statement is: “The LCM instrumentation for this work was provided by a Champlin Foundation grant to the URI College of Pharmacy and College of the Environment and Life Sciences. The cryostat used in this work was provided by funding from an NIH Instrumentation award 1S10OD032209-01. This work was also supported by an internal URI College of the Environment and Life Sciences grant and an external NSF Division of Biological Infrastructure grant 2316390 to HMP. This material is based upon work supported by the National Science Foundation Graduate Research Fellowship Program under Grant No 2146759 to ZD. Any opinions, findings, and conclusions or recommendations expressed in this material are those of the author(s) and do not necessarily reflect the views of the National Science Foundation. There was no additional external funding received for this study.”

Our updated Role of Funder statement is: “The funders had no role in study design, data collection and analysis, decision to publish, or preparation of the manuscript.”

Please contact us should you require any further information.

Sincerely,

Zoe Dellaert and Hollie Putnam

Reviewers' comments (Our responses in blue text):

Reviewer #1: The manuscript by Dellaert and Putnam reports a transcriptomic comparison between oral and aboral territories in apolyps of a reef-building coral species, Pocillopora acuta. This bulk transcriptomic approach complements recent efforts to characterize the cellular repertoire in cnidarians, a group of major interest to understand the origins of bilaterians.

The results from the transcriptomic analysis are robust, well described, well illustrated by quality figures. They are adequately analyzed in terms of functional annotation, with a focus on processes of interest: metabolism, development, response to biotic or abiotic stimuli. As such, they provide a reference for further studies.

As a non-specialist of the field, I have only minor suggestions for improvement:

Experimental aspects:

The work would be valued by direct ISH or HCR-ISH validations of the transcriptomic data, focussing on processes of interest.

We agree that the targeted quantification of expression is the a powerful approach, but given the biological validation we have presented in our work and the point of our paper providing a whole transcriptome view of targeted tissues, the use of direct ISH or HCR-ISH validations of the transcriptomic data would represent a substantial transition of focus and investment of time and money that is beyond the scope of what is available to this work To acknowledge the value and power of direct ISH or HCR-ISH data we have highlighted where our study has the support of multiple studies for biological validation (Lines 661, 698, 711, 723, 729) and we have also added a statement regarding this to the discussion as a recommendation for future work: “Future studies validating these findings with in situ hybridization approaches (e.g., HCR-ISH), particularly for the non-canonical expression of developmental signaling pathways and genes with unexpected tissue-level expression patterns, will greatly advance our understanding of tissue-specific function in corals.” (Line 745)

Minor text modifications:

1. A phylogenetic tree showing the relationships between cnidarians, with the relative position of P. acuta and other species submitted to scRNA-seq analysis or other transcriptomic approach would help the non-specialist reader to have a broader view of data available in this taxon

If the reviewer deems this suitable, we have added the following phylogenetic tree to the supplement (S1 Fig). We do note that the scRNA marker genes referred to in our main text are all from Levy et al. 2021, which is in a closely related species of the same Pocilloporidae family: “, we examined the expression of scRNA-seq marker genes from another Pocilloporid species: S. pistillata [31].” (Line 539).

2. Throughout the manuscript, the authors tend to oversell the spatial resolution brought by their analysis: these data provide a comparison between two spatially distinct tissues. But spatially resolved transcriptomics rely on completely different approaches and provide much more detailed information.

We agree that there are now novel high resolution approaches available at the whole transcriptome level (e.g., spatial transcriptomics and work at the single cell and single nuclei). This is stated in the abstract (Line 47) and we have now added text to reinforce our recommendations for future work in the conclusion: “Sequencing-based methods with higher spatial resolution than the tissue-level work in this paper (e.g., spatial transcriptomics) will be crucial to fully resolve the tissue complexity of these organisms.” (Line 749).

While the novel spatial transcriptomics methods can provide higher resolution and cell-specific information than the approach in our manuscript, our application of Laser Capture Microdissection offers a more affordable alternative for studying the tissues of these organisms, enabling higher replication and broader accessibility to research groups.

The introduction has been revised to not oversell the resolution of LCM (Line 140).

3. line 49 "Corals are complex symbiotic meta-organisms with functionally partitioned tissue architecture,thus integrating tissue-specific processes is essential for improving predictions of responses to climate change stressors and identifying mechanisms underlying resilience": truncated sentence?

We have revised it to read: “Corals are complex symbiotic meta-organisms with functionally partitioned tissue architecture. Thus, integrating tissue-specific processes is essential for improving predictions of responses to climate change stressors and identifying mechanisms underlying their resilience.” (Line 66)

4. line 477 "Both oral and aboral tissues respond to biotic and abiotic stimulus": this is not demonstrated, even if the data support the hypothesis, and it should be reformulated.

We have revised this heading to be more specific: “Both oral and aboral tissues express biotic and abiotic stimulus-response genes” (Line 519)

Reviewer #2: In this manuscript, Dellaert and Putnam investigate genes that are differentially expressed between the oral and aboral sides of the reef-building coral Pocillopora acuta. To do so, they performed RNA sequencing on samples obtained from polyp cryosections using laser capture microdissection. This method is novel in corals, and the resulting data appear to be of high quality, providing a unique dataset for analyzing differential gene expression between the oral and aboral sides of coral polyps. Based on these analyses, the authors draw several interesting conclusions regarding the localization of previously identified cell types and the possible role of Wnt signaling in skeleton formation on the aboral side. I have only a few minor comments that I would like the authors to address:

When analyzing genes that are differentially expressed between the oral and aboral sides of the polyp, the authors rely heavily on Gene Ontology (GO) terms. While this leads to some interesting conclusions, it also results in the discussion of GO terms that seem irrelevant for cnidarians, such as "skin morphogenesis" (L465) and "oviduct epithelium development" (L466), as well as others whose relevance is far from straightforward in cnidarians, including "stimulus involved in sensory perception," "detection of abiotic stimulus," "response to norepinephrine," "cellular response to thyroid hormone stimulus," and "complement activation, classical pathway" (L478–488). I would suggest restricting the discussion to GO terms that are likely, or at least plausibly, applicable to cnidarians.

Because GO terms are primarily derived from model organisms such as humans and C. elegans, some terms can appear inapplicable to cnidarians at face value. However, the underlying genes assigned to these terms often represent deeply conserved evolutionary pathways that are highly relevant to cnidarian biology. For example, cnidarians have sensory cells that respond to touch, light, and chemical cues, and cnidarian innate immunity is crucial to active regulation of endosymbiont populations via digestion and apoptosis induction. These terms mentioned by the reviewer, therefore despite the arguable relevance of their term name, capture biologically meaningful signals in our dataset, even if the human-derived term labels are not literal descriptions of cnidarian processes. Therefore we have maintained all of the significantly enriched GO terms as they identify groups of genes under a common process that are enriched and should be examined for their cnidarian specific biology, not thrown out.

To make this framework explicit, we have added the statement following to the Methods: “We acknowledge that many GO terms are labelled based on mammalian- and human-specific processes, but the specific genes underlying the enrichment of certain terms represent shared evolutionary pathways that are relevant to cnidarian biology. Throughout the text, we use GO terms as tools to identify broad patterns of genes, but all biological interpretation is based on the genes themselves, not the GO term labels.” (Line 344).

In response to the reviewer’s suggestion, we have also edited Figure 3 to replace the term “camera-type eye development” with its parent term “sensory system development,” which more accurately reflects the context of our study. We have indicated this by an * after the term and stated this in the figure caption. Where other term labels appear model-system-specific, we have provided both the label and the parent term label to indicate the broader processes involved (Lines 504, 506).

L473–474: "The top five upregulated genes contributing to these enrichments." It is unclear what "these" refers to. Please clarify.

Thank you for pointing this out! We have revised the sentence to: “The top five upregulated genes contributing to the enrichments of GO terms in of Oral Clusters 1-3 were: Transcription factor Sox2, Peroxidasin homolog pxn-2, Wnt-2b, Double-stranded RNA-specific editase 1, and Dynein axonemal heavy chain 5.” (Line 514)

L537–542: The authors report stronger GO term enrichment on the aboral side but a greater number of differentially expressed genes on the oral side. They conclude that this suggests "transcriptional diversity rather than coordination" on the oral side, whereas the aboral side exhibits "a functionally coordinated expression profile." I do not fully understand what the authors mean by this interpretation. I suggest rewriting this section to make the rationale clearer.

This has been revised to read “While oral tissues showed substantially more upregulated genes than aboral tissue, GO enrichment revealed relatively few oral-enriched functional categories, suggesting that many diverse genes with different functions were upregulated. In contrast, aboral tissues showed fewer upregulated genes, but a larger number of significantly enriched GO terms, indicating that the upregulated genes shared similar functions.” (Line 587)

L471–472: Among the genes enriched on the aboral side of the polyp, the authors identify several genes known to be expressed on the aboral side of cnidarian planulae, including Frizzled5, SFRP1/5, and SFRP3/4, as reported in Ref. 42. Are other conserved aboral genes identified in Ref. 42, such as Six3/6, Hox9–14, and DKK, also present in this dataset? This is an interesting observation that could be further discussed in the Discussion.

We have added a reference to the Wnt signalling expression patterns documented in larvae by Ref. 42 in the Discussion (Line 698). We note that in the supplementary materials of Ref. 42 we are not able to find sufficient information to unambiguously identify gene IDs to search for orthologs based on the shortened gene names used in the text of Ref. 42. We therefore examined the expression of a series of Nematostella Wnt and Hox genes described in Ref. 172 (DuBuc et al., 2018) and found that these overlapped with the genes already discussed in our Results and Discussion; no additional text was added to the Discussion regarding these genes.

Of the additional specific genes from Ref. 42 mentioned by the reviewer:

● Six3/6 was upregulated in aboral tissue but did not meet the cutoff of |LFC| > 1 (log2FoldChange = -0.90, padj = 0.026).

● Dkk1/2/4 was not expressed in our study.

● Hox9-14 does not appear to have a clearly annotated Pocillopora ortholog in Ref. 42, and we were unable to identify a Hox9-14 ortholog in our expressed genes.

Attachment Submitted filename: PLOS_ResponseToReviewers.docx

# Editor report: Decision Letter 1

**DOI:** 10.1371/journal.pone.0358454.r003

**Authors:** Hector Escriva

1 Sep 2026

Spatially resolved gene expression analysis illuminates location-specific functions in the reef-building coral *Pocillopora acuta*

PONE-D-26-21333R1

Dear Dr. Dellaert,

We’re pleased to inform you that your manuscript has been judged scientifically suitable for publication and will be formally accepted for publication once it meets all outstanding technical requirements.

Within one week, you’ll receive an e-mail detailing the required amendments. When these have been addressed, you’ll receive a formal acceptance letter and your manuscript will be scheduled for publication.

An invoice will be generated when your article is formally accepted. Please note, if your institution has a publishing partnership with PLOS and your article meets the relevant criteria, all or part of your publication costs will be covered. Please make sure your user information is up-to-date by logging into Editorial Manager at Editorial Manager® and clicking the ‘Update My Information' link at the top of the page. For questions related to billing, please contact billing support.

If your institution or institutions have a press office, please notify them about your upcoming paper to help maximize its impact. If they’ll be preparing press materials, please inform our press team as soon as possible -- no later than 48 hours after receiving the formal acceptance. Your manuscript will remain under strict press embargo until 2 pm Eastern Time on the date of publication. For more information, please contact onepress@plos.org.

Kind regards,

Hector Escriva, PhD

Academic Editor

PLOS One

Additional Editor Comments (optional):

Reviewers' comments:

# Editor report: Acceptance letter

**DOI:** 10.1371/journal.pone.0358454.r004

**Authors:** Hector Escriva

PONE-D-26-21333R1

PLOS One

Dear Dr. Dellaert,

I'm pleased to inform you that your manuscript has been deemed suitable for publication in PLOS One. Congratulations! Your manuscript is now being handed over to our production team.

At this stage, our production department will prepare your paper for publication. This includes ensuring the following:

* All references, tables, and figures are properly cited

* All relevant supporting information is included in the manuscript submission,

* There are no issues that prevent the paper from being properly typeset

You will receive further instructions from the production team, including instructions on how to review your proof when it is ready. Please keep in mind that we are working through a large volume of accepted articles, so please give us a few days to review your paper and let you know the next and final steps.

Lastly, if your institution or institutions have a press office, please let them know about your upcoming paper now to help maximize its impact. If they'll be preparing press materials, please inform our press team within the next 48 hours. Your manuscript will remain under strict press embargo until 2 pm Eastern Time on the date of publication. For more information, please contact onepress@plos.org.

You will receive an invoice from PLOS for your publication fee after your manuscript has reached the completed accept phase. If you receive an email requesting payment before acceptance or for any other service, this may be a phishing scheme. Learn how to identify phishing emails and protect your accounts at https://explore.plos.org/phishing.

If we can help with anything else, please email us at customercare@plos.org.

Thank you for submitting your work to PLOS One and supporting open access.

Kind regards,

PLOS One Editorial Office Staff

on behalf of

Dr. Hector Escriva

Academic Editor

PLOS One
